# The impacts of anemia burden on clinical outcomes in patients with out‐of‐hospital cardiac arrest

**DOI:** 10.1002/clc.24175

**Published:** 2023-10-24

**Authors:** I‐Wei Ho, Ming‐Jen Kuo, Pai‐Feng Hsu, I‐Hsin Lee, Teh‐Fu Hsu, Yenn‐Jiang Lin, Chin‐Chou Huang

**Affiliations:** ^1^ Division of Cardiology, Department of Medicine Taipei Veterans General Hospital Taipei Taiwan; ^2^ School of Medicine National Yang Ming Chiao Tung University Taipei Taiwan; ^3^ Healthcare and Services Center Taipei Veterans General Hospital Taipei Taiwan; ^4^ Department of Emergency Taipei Veterans General Hospital Taipei Taiwan; ^5^ Heart Rhythm Center Taipei Veterans General Hospital Taipei Taiwan; ^6^ Institute of Pharmacology, School of Medicine National Yang Ming Chiao Tung University Taipei Taiwan

**Keywords:** anemia burden, cardiac arrest, outcome, return of spontaneous circulation

## Abstract

**Background:**

Out‐of‐hospital cardiac arrest (OHCA) has low survival rates, and few patients achieve a desirable neurological outcome. Anemia is common among OHCA patients and has been linked to worse outcomes, but its impact following the return of spontaneous circulation (ROSC) is unclear. This study examines the relationship between anemia burden and clinical outcomes in OHCA patients.

**Hypothesis:**

Higher anemia burden after ROSC may be related to higher mortality and worse neurologic outcomes.

**Methods:**

Patients who experienced OHCA and had ROSC were enrolled retrospectively. Anemia burden was defined as the area under curve from the target hemoglobin level over a 72‐h period after OHCA. Hemoglobin level was measured at 12‐h intervals. The clinical outcomes of the study included mortality and neurological outcomes at Day 30.

**Results:**

The study enrolled 258 nontraumatic OHCA patients who achieved ROSC between January 2017 and December 2021. Among the 162 patients who survived more than 72 h, a higher anemia burden, specifically target hemoglobin levels below 7 (hazard ratio [HR]: 1.129, 95% confidence interval [CI]: 1.013–1.259, *p* = .029), 8 (HR: 1.099, 95% CI: 1.014–1.191, *p* = .021), and 9 g/dL (HR: 1.066, 95% CI: 1.001–1.134, *p* = .046) was associated with higher 30‐day mortality. Additionally, anemia burden with target hemoglobin levels below 7 (HR: 1.129, 95% CI: 1.016–1.248; *p* = .024) and 8 g/dL (HR: 1.088; 95% CI: 1.008–1.174, *p* = .031) was linked to worse neurological outcomes.

**Conclusions:**

Anemia burden predicts 30‐day mortality and neurological outcomes in OHCA patients who survive more than 72 h. Maintaining higher hemoglobin levels within the first 72 h after ROSC may improve short‐term outcomes.

## Graphical abstract

Anemia burden is a predictive factor for 30‐day mortality and worse neurological outcomes in out‐of‐hospital cardiac arrest (OHCA) patients who survive beyond 72 h. Maintaining higher hemoglobin levels after return of spontaneous circulation (ROSC) may potentially improve short‐term outcomes.

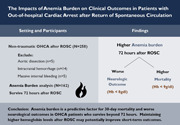

## 1 INTRODUCTION

Cardiac arrest is a critical medical emergency that can result in significant morbidity and mortality.^1^ Despite significant advancements in resuscitation techniques, survival rates after out‐of‐hospital cardiac arrest (OHCA) remain low.^2^, ^3^ Only a small percentage of OHCA survivors achieve a desirable neurological outcome.^4^ Due to these challenges, these patients have many unmet needs, and further studies are necessary to improve outcomes.

Anemia is a common comorbidity in patients with cardiovascular diseases and has been identified as an independent predictor of adverse outcomes in various settings.^5^, ^6^, ^7^ Previous studies have shown that lower initial hemoglobin levels are associated with higher mortality and worse neurological outcomes in OHCA patients after return of spontaneous circulation (ROSC).^8^, ^9^ However, its universal validity remains uncertain. Moreover, the cumulative impact of post‐ROSC anemia on the clinical outcomes of these patients remains unexplored. In previous relevant studies focusing on OHCA patients after ROSC, a discernible correlation with prognosis during the first 72 h has been observed.^10^, ^11^ Therefore, we hypothesize that anemia within the first 72‐h period might have the most significant influence on clinical outcomes of these patients.

The effectiveness of blood transfusion as a treatment for OHCA patients with low hemoglobin levels is uncertain due to a lack of supporting data. Clinical trials that support maintaining lower hemoglobin thresholds for transfusion have often excluded patients with severe ischemic events, such as myocardial infarction.^12^, ^13^, ^14^ Studies on whether to transfuse patients with cardiovascular disease have produced conflicting results.^15^ The optimal hemoglobin threshold for transfusion in this population is still unclear, and more research is needed to determine its effectiveness and safety.

This study aims to investigate the relationship between anemia burden, cumulative effects of anemia within the first 72‐h period, and clinical outcomes in OHCA patients after ROSC. Understanding this relationship could provide valuable insights for this patient population's risk stratification and management strategies. The findings from this study may have important implications for improving outcomes in OHCA patients after ROSC and may inform the use of blood transfusion to mitigate the impact of anemia burden on clinical outcomes.

## 2 METHODS

### 2.1 Study subjects and study design

The study was conducted at Taipei Veterans General Hospital, a national medical center in Taiwan. The eligibility criteria for patient enrollment included being at least 20 years old, having experienced OHCA due to nontraumatic causes, and having achieved ROSC after cardiopulmonary resuscitation (CPR) between January 1, 2017, and December 31, 2022. Patients with active bleeding were excluded, including aortic dissection, intracranial hemorrhage, and massive internal bleeding.

Our study comprised two parts. In the first part, we aimed to confirm the association between initial hemoglobin levels and clinical outcomes. In the second part, we aimed to investigate the relationship between anemia burden and clinical outcomes in patients who survived for more than 72 h.

The study protocol received approval from the Ethics Committee of Taipei Veterans General Hospital (approval number: 2022‐09‐009AC). Given the study's retrospective nature, the need for informed consent was waived. The study was conducted in accordance with the principles of the Declaration of Helsinki.

### 2.2 Study variables and patient data

This retrospective study collected data from the Taipei Veterans General Hospital database. Baseline characteristics, including age, gender, comorbidities, and variables derived from the Utstein template, were collected.^16^ All patients were admitted to the critical care unit after ROSC, and laboratory tests were performed at least once daily. Data from the first 72 h following ROSC were collected.

Comorbidities included hypertension, diabetes mellitus (DM), end‐stage renal disease (ESRD), liver cirrhosis, chronic obstructive pulmonary disease (COPD), heart failure, arrhythmia, coronary artery disease (CAD), old myocardial infarction (MI), peripheral arterial occlusive disease (PAOD), and cancer.

### 2.3 Anemia burden

Anemia burden was defined as the area under curve from the target hemoglobin level over a 72‐h after OHCA period. We measured hemoglobin level at 12‐h intervals. In cases where data were missing, we utilized an interpolation method based on 24‐h hemoglobin levels to estimate the value.

### 2.4 Clinical outcomes

The clinical outcomes of the study included mortality and neurological outcomes at Day 30. Good neurological outcomes were defined as a Cerebral Performance Category Scale (CPC scale) score of 1 or 2.

### 2.5 Statistical analyses

The characteristics of the study participants were summarized using descriptive statistics. Quantitative variables are expressed as mean ± standard deviation, and categorical variables are expressed as numbers (percentages). Parametric continuous data between separate groups were compared using the unpaired Student's *t* test, while nonparametric data were compared using the Mann–Whitney test. Cox proportional hazards regression analysis was performed, and adjusted hazard ratios (HRs) with 95% confidence intervals (CIs) were estimated after adjusting for potential confounding factors. Statistical significance was inferred at a two‐sided *p* < .05. We conducted the statistical analyses using SPSS software (version 21.0; SPSS Inc.).

## 3 RESULTS

### 3.1 Baseline characteristics of all patients

This study included 258 nontraumatic OHCA patients who achieved ROSC between January 2017 and December 2021. After excluding 24 patients with active bleeding due to aortic dissection (*n* = 5), intracranial hemorrhage (*n* = 14), and massive internal bleeding (*n* = 5), 234 patients were enrolled for further analysis (Supporting Information: Figure 1).

Among the 234 patients, 104 patients survived and 130 patients who did not survive. When comparing surviving patients, those who did not survive were older (*p* = .032), less likely to receive bystander CPR (*p* < .001), less likely to have a shockable rhythm (*p* < .001), more likely to have a “do not resuscitate” (DNR) order after ROSC (*p* < .001), and more commonly use of extracorporeal membrane oxygenation (ECMO) (*p* = .045). The causes of OHCA were significantly different between the two groups, with a lower proportion of cardiac causes (*p* = .005) and a higher proportion of septic shock (*p* < .001) in those who did not survive. In terms of comorbidities, DM (*p* = .016), ESRD (*p* = .046), heart failure (*p* = .046), and arrhythmia (*p* = .006) were more prevalence in those who did not survive. Surviving patients had higher initial hemoglobin levels than those who did not survive (13.0 ± 3.0 vs. 11.2 ± 2.7, *p* < .001) (Table 1).

**Table 1:** **Baseline characteristics of all patients (*n*** = **234)**.

	Survival (*n* = 104)	Mortality (*n* = 130)	*p* Value	CPC 1–2 (*n* = 54)	CPC 3–5 (*n* = 180)	*p* Value
Age, years	65.2 ± 15.6	69.8 ± 16.7	.032	59.0 ± 15.7	70.3 ± 15.7	<.001
Male, *n* (%)	64 (61.5%)	77 (59.2%)	.720	29 (53.7%)	112 (62.2%)	.262
Bystander CPR, *n* (%)	57 (54.8%)	38 (29.2%)	<.001	34 (63.0%)	61 (33.9%)	<.001
Shockable rhythm, *n* (%)	50 (48.1%)	29 (22.3%)	<.001	38 (70.4%)	41 (22.8%)	<.001
DNR after ROSC, *n* (%)	25 (24.0%)	78 (60.0%)	<.001	5 (9.3%)	98 (54.4%)	<.001
ECMO, *n* (%)	14 (13.5%)	31 (23.8%)	.045	6 (11.1%)	39 (21.7%)	.084
TTM, *n* (%)	38 (36.5%)	37 (28.5%)	.188	20 (37.0%)	55 (30.6%)	.371
Causes of OHCA						
Asphyxia, *n* (%)	22 (21.2%)	35 (26.9%)	.307	4 (7.4%)	53 (29.4%)	.001
Cardiac causes, *n* (%)	68 (65.4%)	61 (46.9%)	.005	46 (85.2%)	83 (46.1%)	<.001
Pulmonary embolism, *n* (%)	4 (3.8%)	1 (0.8%)	.123	0 (0.0%)	5 (2.8%)	.266
Drug poisoning, *n* (%)	1 (1.0%)	4 (3.1%)	.262	1 (1.9%)	4 (2.2%)	.674
Hyperkalemia, *n* (%)	4 (3.8%)	7 (5.4%)	.409	2 (3.7%)	9 (5.0%)	.514
Septic shock, *n* (%)	2 (1.9%)	21 (16.2%)	<.001	0 (0.0%)	23 (12.8%)	.006
Other medical cause, *n* (%)	3 (2.9%)	1 (0.8%)	.232	1 (1.9%)	3 (1.7%)	.653
Comorbidities						
HTN, *n* (%)	58 (55.8%)	78 (60.0%)	.515	30 (55.6%)	106 (58.9%)	.663
DM, *n* (%)	29 (27.9%)	56 (43.1%)	.016	14 (25.9%)	71 (39.4%)	.070
ESRD, *n* (%)	9 (8.7%)	23 (17.7%)	.046	6 (11.1%)	26 (14.4%)	.532
Liver cirrhosis, *n* (%)	0 (0.0%)	2 (1.5%)	.308	0 (0.0%)	2 (1.1%)	.591
COPD, *n* (%)	4 (3.8%)	3 (2.3%)	.378	3 (5.6%)	4 (2.2%)	.202
Heart failure, *n* (%)	9 (8.7%)	23 (17.7%)	.046	2 (3.7%)	30 (16.7%)	.015
Arrhythmia, *n* (%)	5 (4.8%)	21 (16.2%)	.006	1 (1.9%)	25 (13.9%)	.014
CAD/Old MI, *n* (%)	23 (22.1%)	26 (20.0%)	.693	11 (20.4%)	38 (21.1%)	.907
Old CVA, *n* (%)	14 (13.5%)	18 (13.8%)	.932	4 (7.4%)	28 (15.6%)	.231
PAOD, *n* (%)	5 (4.8%)	6 (4.6%)	.591	4 (7.4%)	7 (3.9%)	.126
Cancer, *n* (%)	14 (13.5%)	12 (9.2%)	.306	7 (13.0%)	19 (10.6%)	.622
Hgb (initial), mg/dL	13.0 ± 3.0	11.2 ± 2.7	<.001	13.7 ± 2.9	11.5 ± 2.7	<.001
Follow up, days	22.7 ± 8.3	7.2 ± 8.1	<.001	17.9 ± 7.8	13.0 ± 11.9	.005

Abbreviations: CAD, coronary artery disease; COPD, chronic obstructive pulmonary disease; CPR, cardiopulmonary resuscitation; CVA, cerebrovascular accident; DM, diabetes mellitus; DNR, do not resuscitate; ECMO, extracorporeal membrane oxygenation; ESRD, end‐stage renal disease; Hgb, hemoglobin; HTN, hypertension; MI, myocardial infarction; OHCA, out‐of‐hospital cardiac arrest; PAOD, peripheral arterial occlusive disease; ROSC, return of spontaneous circulation; TTM, targeted temperature management.

Among the 234 patients, 54 had good neurological outcomes (CPC: 1–2), and 180 had worse neurological outcomes (CPC: 3–5). When comparing to those with good neurological outcomes, patients with worse neurological outcomes were significantly older (*p* < .001), less likely to receive bystander CPR (*p* < .001), less likely to have a shockable rhythm (*p* < .001), and more likely to have a DNR order after ROSC (*p* < .001). The causes of OHCA were significantly different between the two groups, with a lower proportion of cardiac causes (*p* < .001), a higher proportion of asphyxia (*p* = .001), and a higher proportion of septic shock (*p* = .006) in patients with worse neurological outcomes. Comorbidities such as heart failure (*p* = .015) and arrhythmia (*p* = .014) were more prevalent in those with worse neurological outcomes. Patients with good neurological outcomes had higher initial hemoglobin levels than those with worse neurological outcomes (13.7 ± 2.9 vs. 11.5 ± 2.7, *p* < .001) (Table 1).

### 3.2 Independent predictors of 30‐day mortality and neurological outcome in all patients

Cox multivariate regression analysis showed that a DNR order after ROSC (HR = 2.169; CI: 1.426–3.298, *p* < .001) and ECMO (HR = 2.336; CI: 1.403–3.992, *p* = .001) were significant independent predictors of 30‐day mortality. However, initial hemoglobin level was not found to be a significant independent predictor of 30‐day mortality (Table 2).

**Table 2:** **Independent predictors of 30‐day mortality and neurologic outcomes in all patients (*n*** = **234)**.

	HR	(95% CI)	*p* Value
Mortality			
Age, years	0.994	(0.981–1.008)	.412
Gender (male vs. female)	0.912	(0.623–1.336)	.636
Bystander CPR (yes vs. no)	0.663	(0.439–1.001)	.051
Shockable rhythm (yes vs. no)	0.574	(0.336–0.982)	.043
DNR after ROSC (yes vs. no)	2.169	(1.426–3.298)	<.001
ECMO (yes vs. no)	2.366	(1.403–3.992)	.001
Cardiac cause (yes vs. no)	0.972	(0.598–1.579)	.908
Septic shock (yes vs. no)	1.496	(0.869–2.577)	.146
DM (yes vs. no)	1.260	(0.858–1.849)	.239
ESRD (yes vs. no)	1.371	(0.820–2.294)	.229
Heart failure (yes vs. no)	0.997	(0.616–1.613)	.989
Arrhythmia (yes vs. no)	1.503	(0.902–2.504)	.118
Hgb (initial), mg/dL	0.962	(0.896–1.032)	.284
Neurologic outcomes			
Age, years	0.998	(0.986–1.010)	.718
Male (yes vs. no)	0.934	(0.669–1.305)	.690
Bystander CPR (yes vs. no)	0.853	(0.609–1.193)	.353
Shockable rhythm (yes vs. no)	0.750	(0.475–1.182)	.215
DNR after ROSC (yes vs. no)	1.723	(1.222–2.430)	.002
Asphyxia (yes vs. no)	0.999	(0.590–1.692)	.997
Cardiac cause (yes vs. no)	1.031	(0.589–1.805)	.914
Septic shock (yes vs. no)	1.772	(0.969–3.242)	.063
Heart failure (yes vs. no)	1.087	(0.724–1.632)	.689
Arrhythmia (yes vs. no)	1.461	(0.936–2.280)	.095
Hgb (initial), mg/dL	0.971	(0.918–1.028)	.314

Abbreviations: CI, confidence interval; CPR, cardiopulmonary resuscitation; DM, diabetes mellitus; DNR, do not resuscitate; ECMO, extracorporeal membrane oxygenation; ESRD, end‐stage renal disease; Hgb, hemoglobin; HR, hazard ratio; ROSC, return of spontaneous circulation.

Cox multivariate regression analysis showed that a DNR order after ROSC (HR = 1.723, CI: 1.222–2.430, *p* < .001) was found to be a significant independent predictor of 30‐day worse neurological outcomes. However, similar to the mortality analysis, initial hemoglobin levels were not found to be significant independent predictors of 30‐day neurological outcomes (Table 2).

### 3.3 Baseline characteristics of patients survived for more than 72 h

For the second part of the analysis, 72 patients who died within 72 h of ROSC were excluded. Finally, a total of 162 patients were analyzed. The detail information was presented in Table 3.

**Table 3:** **Baseline characteristics of patients survived for more than 72 h (*n*** = **162)**.

	Survival (*n* = 103)	Mortality (*n* = 59)	*p* Value	CPC 1–2 (*n* = 53)	CPC 3–5 (*n* = 109)	*p* Value
Age, years	65.2 ± 15.7	69.7 ± 15.6	.080	58.9 ± 15.8	70.7 ± 14.3	<.001
Male, *n* (%)	63 (61.2%)	34 (57.6%)	.658	28 (52.8%)	69 (63.3%)	.202
Bystander CPR, *n* (%)	56 (54.4%)	19 (32.2%)	.006	33 (62.3%)	42 (38.5%)	.004
Shockable rhythm, *n* (%)	49 (47.6%)	15 (25.4%)	.006	37 (69.8%)	27 (24.8%)	<.001
DNR after ROSC, *n* (%)	25 (24.3%)	36 (61.0%)	<.001	5 (9.4%)	56 (51.4%)	<.001
ECMO, *n* (%)	14 (13.6%)	14 (23.7%)	.101	6 (11.3%)	22 (20.2%)	.162
TTM, *n* (%)	38 (36.9%)	20 (33.9%)	.702	20 (37.7%)	38 (34.9%)	.720
Causes of OHCA						
Asphyxia, *n* (%)	22 (21.4%)	16 (27.1%)	.405	4 (7.5%)	34 (31.2%)	.001
Cardiac causes, *n* (%)	67 (65.0%)	27 (45.8%)	.017	45 (84.9%)	49 (45.0%)	<.000
Pulmonary embolism, *n* (%)	4 (3.9%)	0 (0.0%)	.160	0 (0.0%)	4 (3.7%)	.201
Drug poisoning, *n* (%)	1 (1.0%)	2 (3.4%)	.300	1 (1.9%)	2 (1.8%)	.698
Hyperkalemia, *n* (%)	4 (3.9%)	3 (5.1%)	.502	2 (3.8%)	5 (4.6%)	.585
Septic shock, *n* (%)	2 (1.9%)	10 (16.9%)	.001	0 (0.0%)	12 (11.0%)	.007
Other medical cause, *n* (%)	3 (2.9%)	1 (1.7%)	.537	1 (1.9%)	3 (2.8%)	.604
Comorbidities						
HTN, *n* (%)	58 (56.3%)	37 (62.7%)	.426	30 (56.6%)	65 (59.6%)	.713
DM, *n* (%)	29 (28.2%)	29 (49.2%)	.007	14 (26.4%)	44 (40.4%)	.082
ESRD, *n* (%)	9 (8.7%)	10 (16.9%)	.118	6 (11.3%)	13 (11.9%)	.910
Cirrhosis, *n* (%)	0 (0.0%)	0 (0.0%)	‐	0 (0.0%)	0 (0.0%)	‐
COPD, *n* (%)	4 (3.9%)	1 (1.7%)	.399	3 (5.7%)	2 (1.8%)	.198
Heart failure, *n* (%)	9 (8.7%)	7 (11.9%)	.521	2 (3.8%)	14 (12.8%)	.069
Arrhythmia, *n* (%)	5 (4.9%)	9 (15.3%)	.023	1 (1.9%)	13 (11.9%)	.025
CAD/Old MI, *n* (%)	23 (22.3%)	8 (13.6%)	.172	11 (20.8%)	20 (18.3%)	.715
Old CVA, *n* (%)	14 (13.6%)	7 (11.9%)	.753	4 (7.5%)	17 (15.6%)	.152
PAOD, *n* (%)	5 (4.9%)	2 (3.4%)	.498	4 (7.5%)	3 (2.8%)	.159
Cancer, *n* (%)	14 (13.6%)	6 (10.2%)	.524	7 (13.2%)	13 (11.9%)	.816
Hgb (initial), mg/dL	12.9 ± 3.0	11.2 ± 2.9	.001	13.7 ± 3.0	11.6 ± 2.9	<.001
Hgb (12 h), mg/dL	12.1 ± 2.8	10.7 ± 2.7	.002	12.5 ± 2.7	11.2 ± 2.7	.005
Hgb (24 h), mg/dL	11.5 ± 2.6	10.2 ± 2.5	.001	11.9 ± 2.4	10.6 ± 2.6	.003
Hgb (36 h), mg/dL	11.4 ± 2.5	10.0 ± 2.3	.000	11.7 ± 2.4	10.5 ± 2.5	.004
Hgb (48 h), mg/dL	11.2 ± 2.1	9.7 ± 2.0	<.001	11.4 ± 2.1	10.3 ± 2.1	.001
Hgb (60 h), mg/dL	10.9 ± 2.1	9.5 ± 1.8	<.001	11.1 ± 1.9	10.0 ± 2.1	.001
Hgb (72 h), mg/dL	10.8 ± 1.9	9.1 ± 2.2	<.001	10.8 ± 1.7	9.8 ± 2.3	.003
Hgb (average), mg/dL	11.6 ± 2.2	10.1 ± 2.0	<.001	11.9 ± 2.2	10.6 ± 2.2	.001
Anemia burden 7, day × g/dL	0.1 ± 0.7	0.6 ± 2.4	.063	0.0 ± 0.1	0.4 ± 1.9	.156
Anemia burden 8, day × g/dL	0.3 ± 1.0	1.3 ± 3.4	.005	0.2 ± 0.5	0.9 ± 2.7	.040
Anemia burden 9, day × g/dL	1.1 ± 2.2	3.2 ± 4.9	<.001	0.8 ± 1.8	2.4 ± 4.1	.007
Anemia burden 10, day × g/dL	2.9 ± 4.4	6.5 ± 6.9	<.001	2.3 ± 4.1	5.2 ± 6.1	.002
Anemia burden 11, day × g/dL	5.5 ± 7.0	10.9 ± 9.0	<.001	4.4 ± 6.7	9.0 ± 8.4	.001
Follow‐up, days	22.9 ± 8.0	14.1 ± 7.5	<.001	18.2 ± 7.5	20.5 ± 9.4	.127

Abbreviations: CAD, coronary artery disease; COPD, chronic obstructive pulmonary disease; CPR, cardiopulmonary resuscitation; CVA, cerebrovascular accident; DM, diabetes mellitus; DNR, do not resuscitate; ECMO, extracorporeal membrane oxygenation; ESRD, end‐stage renal disease; Hgb, hemoglobin; HTN, hypertension; MI, myocardial infarction; OHCA, out‐of‐hospital cardiac arrest; PAOD, peripheral arterial occlusive disease; ROSC, return of spontaneous circulation; TTM, targeted temperature management.

In the survival and good neurological outcomes groups, both the initial hemoglobin level and hemoglobin levels every 12 h up to 72 h were significantly higher compared to the mortality and worse neurological outcomes groups (Table 3).

### 3.4 Anemia burden and 30‐day mortality in patients surviving more than 72 h

The group of patients who experienced mortality demonstrated a significantly higher anemia burden, as indicated by target hemoglobin level below 8 (*p* = .005), 9 (*p* < .001), 10 (*p* < .001), and 11 g/dL (*p* < .001) compared to the group of patients who survived (Table 3).

Cox multivariate regression analysis showed that anemia burden with target hemoglobin levels below 7 (HR: 1.129; 95% CI: 1.013–1.259; *p* = .029), 8 (HR: 1.099; 95% CI: 1.014–1.191; *p* = .021), and 9 g/dL (HR: 1.066; 95% CI: 1.001–1.134; *p* = .046) were independently associated with 30‐day mortality. However, target hemoglobin levels below 10 and 11 g/dL were not significantly associated with mortality (Table 4).

**Table 4:** **Independent predictor of 30‐day mortality in patients surviving more than 72 h (*n*** = **162)**.

	HR	(95% CI)	*p* Value
Age, year	0.982	(0.961–1.002)	.077
Male (yes vs. no)	0.706	(0.402–1.238)	.224
Bystander CPR (yes vs. no)	0.636	(0.345–1.170)	.146
Shockable rhythm (yes vs. no)	0.980	(0.444–2.164)	.960
DNR after ROSC (yes vs. no)	2.758	(1.518–5.010)	.001
Cardiac cause (yes vs. no)	0.811	(0.385–1.707)	.581
Septic shock (yes vs. no)	2.648	(1.232–5.690)	.013
DM (yes vs. no)	2.034	(1.183–3.499)	.010
Arrhythmia (yes vs. no)	2.250	(1.034–4.895)	.041
Anemia burden 7, day × g/dL	1.129	(1.013–1.259)	.029

	**HR**	**(95% CI)**	*p* Value
Age, year	0.982	(0.962–1.002)	.076
Male (yes vs. no)	0.678	(0.385–1.197)	.180
Shockable rhythm (yes vs. no)	0.977	(0.443–2.153)	.954
Bystander CPR (yes vs. no)	0.639	(0.346–1.180)	.152
DNR after ROSC (yes vs. no)	2.732	(1.499–4.977)	.001
Cardiac cause (yes vs. no)	0.863	(0.411–1.811)	.696
Septic shock (yes vs. no)	2.684	(1.249–5.769)	.011
DM (yes vs. no)	1.910	(1.099–3.318)	.022
Arrhythmia (yes vs. no)	2.247	(1.034–4.881)	.041
Anemia burden 8, day × g/dL	1.099	(1.014–1.191)	.021

	**HR**	**(95% CI)**	*p* Value
Age, year	0.981	(0.961–1.001)	.058
Male (yes vs. no)	0.680	(0.386–1.199)	.182
Bystander CPR (yes vs. no)	0.668	(0.364–1.228)	.194
Shockable rhythm (yes vs. no)	1.016	(0.464–2.225)	.969
DNR after ROSC (yes vs. no)	2.851	(1.562–5.203)	.001
Cardiac cause (yes vs. no)	0.906	(0.431–1.908)	.796
Septic shock (yes vs. no)	2.502	(1.161–5.393)	.019
DM (yes vs. no)	1.785	(1.000–3.187)	.050
Arrhythmia (yes vs. no)	2.225	(1.026–4.822)	.043
Anemia burden 9, day × g/dL	1.066	(1.001–1.134)	.046

	**HR**	**(95% CI)**	*p* Value
Age, year	0.979	(0.960–1.000)	.046
Male (yes vs. no)	0.696	(0.397–1.220)	.206
Bystander CPR (yes vs. no)	0.671	(0.366–1.232)	.199
Shockable rhythm (yes vs. no)	1.048	(0.482–2.278)	.905
DNR after ROSC (yes vs. no)	2.947	(1.607–5.405)	<.001
Cardiac cause (yes vs. no)	0.909	(0.431–1.915)	.802
Septic shock (yes vs. no)	2.387	(1.101–5.176)	.028
DM (yes vs. no)	1.749	(0.966–3.164)	.065
Arrhythmia (yes vs. no)	2.225	(1.027–4.822)	.043
Anemia burden 10, day × g/dL	1.043	(0.996–1.092)	.075

	**HR**	**(95% CI)**	*p* Value
Age, year	0.978	(0.959–0.999)	.038
Male (yes vs. no)	0.708	(0.405–1.237)	.225
Bystander CPR (yes vs. no)	0.667	(0.363–1.224)	.191
Shockable rhythm (yes vs. no)	1.059	(0.489–2.296)	.884
DNR after ROSC (yes vs. no)	3.004	(1.632–5.528)	<.001
Cardiac cause (yes vs. no)	0.907	(0.430–1.911)	.797
Septic shock (yes vs. no)	2.318	(1.064–5.048)	.034
DM (yes vs. no)	1.749	(0.970–3.156)	.063
Arrhythmia (yes vs. no)	2.253	(1.038–4.890)	.040
Anemia burden 11, day × g/dL	1.032	(0.997–1.068)	.077

Abbreviations: CI, confidence interval; CPR, cardiopulmonary resuscitation; DM, diabetes mellitus; DNR, do not resuscitate; HR, hazard ratio; ROSC, return of spontaneous circulation.

### 3.5 Anemia burden and neurological outcome in patients surviving more than 72 h

Patients with worse neurological outcomes had higher anemia burden with target hemoglobin levels below 8 (*p* = .040), 9 (*p* = .007), 10 (*p* = .002), and 11 g/dL (*p* = .001) than patients with good neurological outcomes (Table 3).

Cox multivariate regression analysis showed that anemia burden with target hemoglobin levels below 7 (HR: 1.129, 95% CI: 1.016–1.248; *p* = .024) and 8 g/dL (HR: 1.088, 95% CI: 1.008–1.174; *p* = .031) were independently associated with worse neurological outcomes (Table 5). However, anemia burden with target levels below 9, 10, and 11 g/dL were not significantly associated with neurological outcomes (Table 5).

**Table 5:** **Independent predictor of neurologic outcomes in patients surviving more than 72 h (*n*** = **162)**.

	HR	(95% CI)	*p* Value
Age, year	0.998	(0.982–1.014)	.802
Male (yes vs. no)	0.847	(0.548–1.310)	.455
Bystander CPR (yes vs. no)	1.005	(0.654–1.545)	.981
Shockable rhythm (yes vs. no)	0.827	(0.447–1.533)	.547
DNR after ROSC (yes vs. no)	1.993	(1.287–3.085)	.002
Asphyxia (yes vs. no)	0.893	(0.457–1.743)	.740
Cardiac cause (yes vs. no)	0.803	(0.386–1.671)	.558
Septic shock (yes vs. no)	1.972	(0.897–4.336)	.091
Arrhythmia (yes vs. no)	1.518	(0.817–2.820)	.187
Anemia burden 7, day × g/dL	1.126	(1.016–1.248)	.024

	**HR**	**(95% CI)**	*p* Value
Age, year	0.997	(0.982–1.013)	.747
Male (yes vs. no)	0.849	(0.548–1.314)	.462
Bystander CPR (yes vs. no)	1.021	(0.662–1.575)	.924
Shockable rhythm (yes vs. no)	0.855	(0.465–1.573)	.614
DNR after ROSC (yes vs. no)	1.999	(1.289–3.100)	.002
Asphyxia (yes vs. no)	0.881	(0.450–1.726)	.712
Cardiac cause (yes vs. no)	0.811	(0.390–1.688)	.576
Septic shock (yes vs. no)	1.968	(0.893–4.339)	.093
Arrhythmia (yes vs. no)	1.529	(0.823–2.841)	.179
Anemia burden 8, day × g/dL	1.088	(1.008–1.174)	.031

	**HR**	**(95% CI)**	*p* Value
Age, year	0.997	(0.981–1.012)	.669
Male (yes vs. no)	0.865	(0.558–1.340)	.516
Bystander CPR (yes vs. no)	1.034	(0.668–1.602)	.881
Shockable rhythm (yes vs. no)	0.885	(0.483–1.619)	.691
DNR after ROSC (yes vs. no)	2.034	(1.307–3.165)	.002
Asphyxia (yes vs. no)	0.884	(0.450–1.738)	.721
Cardiac cause (yes vs. no)	0.815	(0.392–1.693)	.583
Septic shock (yes vs. no)	1.930	(0.868–4.291)	.107
Arrhythmia (yes vs. no)	1.513	(0.815–2.808)	.189
Anemia burden 9, day × g/dL	1.039	(0.982–1.099)	.182

	**HR**	**(95% CI)**	*p* Value
Age, year	0.997	(0.981–1.013)	.676
Male (yes vs. no)	0.873	(0.564–1.353)	.544
Bystander CPR (yes vs. no)	1.017	(0.657–1.573)	.941
Shockable rhythm (yes vs. no)	0.898	(0.491–1.644)	.728
DNR after ROSC (yes vs. no)	2.033	(1.304–3.168)	.002
Asphyxia (yes vs. no)	0.886	(0.452–1.740)	.726
Cardiac cause (yes vs. no)	0.807	(0.389–1.673)	.564
Septic shock (yes vs. no)	1.945	(0.872–4.338)	.104
Arrhythmia (yes vs. no)	1.517	(0.817–2.816)	.187
Anemia burden 10, day × g/dL	1.017	(0.981–1.054)	.363

	**HR**	**(95% CI)**	*p* Value
Age, year	0.996	(0.980–1.013)	.660
Male (yes vs. no)	0.878	(0.566–1.361)	.560
Bystander CPR (yes vs. no)	1.010	(0.654–1.562)	.963
Shockable rhythm (yes vs. no)	0.903	(0.493–1.653)	.740
DNR after ROSC (yes vs. no)	2.037	(1.306–3.176)	.002
Asphyxia (yes vs. no)	0.887	(0.452–1.740)	.727
Cardiac cause (yes vs. no)	0.805	(0.388–1.668)	.559
Septic shock (yes vs. no)	1.939	(0.868–4.331)	.106
Arrhythmia (yes vs. no)	1.527	(0.822–2.836)	.181
Anemia burden 11, day × g/dL	1.011	(0.986–1.037)	.395

Abbreviations: CI, confidence interval; CPR, cardiopulmonary resuscitation; DNR, do not resuscitate; HR, hazard ratio; ROSC, return of spontaneous circulation.

## 4 DISCUSSION

As far as we know, this study is the first to indicate that anemia burden within the first 72 h following ROSC may negatively impact both mortality and neurological outcomes in OHCA patients. Maintaining hemoglobin levels higher than 9 g/L after ROSC could potentially improve mortality, while levels higher than 8 g/L may improve neurological outcomes.

Previous studies have investigated the link between initial hemoglobin levels and postcardiac arrest neurological outcomes, but our study has several strengths.^9^, ^15^, ^16^, ^17^, ^18^ We not only focused on initial hemoglobin levels, but recorded hemoglobin data within the first 72 h, and we compared clinical outcomes, including mortality and neurological outcomes. We aimed to identify hemoglobin values indicating anemia burden rather than relying on associations. Our findings were consistent with previous studies, as we observed a positive correlation between higher hemoglobin levels upon hospital arrival and favorable 30‐day neurological outcomes in univariate analysis. However, this benefit was not statistically significant in our multivariate analysis, suggesting that initial hemoglobin levels may not strongly influence neurological outcomes. Therefore, we hypothesized that sustained anemia burden after ROSC may be a more critical factor in determining neurological outcomes. Our study found a significant association between higher anemia burden, defined as hemoglobin levels below 7 and 8 g/dL, during the first 72 h after cardiac arrest, and worse 30‐day neurological outcomes. These results suggest that maintaining hemoglobin levels of at least 8 g/dL during the early postcardiac arrest period may have a beneficial effect on improving neurological outcomes in nontraumatic OHCA patients.

Low hemoglobin levels after OHCA have been widely discussed in the literature as associated with worse neurological outcomes, as has been widely discussed in the literature.^9^, ^17^, ^18^, ^19^, ^20^ However, there has been less focus on the relationship between hemoglobin levels and mortality in OHCA patients. Erol et al. reported that the mortality group had significantly lower hemoglobin values at admission to the emergency department than the survival group.^8^ Similarly, our study found a link between lower initial hemoglobin levels and higher mortality rates. However, this association lost significance upon conducting multivariate analysis, possibly due to the modest effect of initial hemoglobin and other variables affecting mortality. Our study revealed a significant association between increased mortality rates and a higher anemia burden with target hemoglobin level below 7, 8, and 9 g/dL in the first 72 h after cardiac arrest. Our findings suggest that monitoring and managing subsequent hemoglobin levels in the days after OHCA may play a vital role in determining patient mortality.

It is possible that anemia merely indicates more severe illness or less favorable cardiac arrest etiology. In a large study of over 17 000 elders with normal renal function, anemia was associated with an increased risk of mortality and hospitalization, even after adjusting for age, sex, and chronic disease.^21^ Furthermore, patients with lower hemoglobin levels may experience worse outcomes in chronic diseases such as congestive heart failure, COPD, and chronic kidney disease.^22^, ^23^, ^24^ Pre‐existing anemia is also a predictor of worse outcomes in patients undergoing elective surgery, acute MI, and patients in the critical care unit, including failure of liberation from mechanical ventilation and increased risk of death.^5^, ^6^, ^25^ Therefore, monitoring and managing hemoglobin levels in OHCA patients is important for prognostic purposes and identifying potential underlying conditions that may impact patient outcomes.

Although blood transfusion may be a potential treatment option to improve outcomes in postcardiac arrest patients with low hemoglobin levels, there is a lack of data supporting its use. Clinical trials that support the conservative approach of maintaining lower hemoglobin thresholds for transfusion have typically excluded patients with severe ischemic events such as MI.^12^, ^13^, ^14^, ^15^ The decision of whether to transfuse patients with cardiovascular disease has yielded conflicting results.^15^ The recent REALITY trial showed that a restrictive transfusion threshold (hemoglobin of ≤8 g/dL) in individuals with acute MI may be acceptable.^26^ Despite this, some studies have included hemoglobin thresholds of 9–10 mg/dL as part of postarrest care bundles.^27^, ^28^ However, the optimal hemoglobin threshold for transfusion in this population remains unclear, and further studies are needed to evaluate its efficacy and safety.

Further research is needed to explore this relationship in greater detail and to develop effective interventions to optimize hemoglobin levels in these patients. Specifically, future studies should focus on investigating the association between hemoglobin levels and both neurological outcomes and mortality in OHCA patients and exploring the potential impact of interventions to improve hemoglobin levels on patient outcomes. Ultimately, a better understanding of the relationship between hemoglobin levels and outcomes in OHCA patients could guide the development of more effective treatment strategies and improve patient outcomes in this population.

### 4.1 Study limitations

Our study had some limitations. First, it is a retrospective, observational study conducted at a single center, which limits the generalizability of the findings. Second, the possibility that anemia may be a marker of underlying disease severity cannot be completely ruled out. However, including multiple risk factors in the analyses helped minimize this possibility. Third, despite having at least one daily hemoglobin measurement, missing data were still present, requiring the use of interpolation methods. Fourth, due to the lack of blood transfusion records, indirect observation of the effects of blood transfusion through monitoring hemoglobin levels every 12 h was employed. Despite these limitations, the study contributes to understanding the relationship between hemoglobin levels and outcomes following cardiac arrest. Future prospective studies are needed to confirm these findings and better evaluate the impact of anemia and transfusion on outcomes in this patient population.

## 5 CONCLUSIONS

Our study suggests that anemia burden with target hemoglobin levels below 7, 8, and 9 g/dL are important predictors of 30‐day mortality, and target levels below 7 and 8 g/dL are important predictors of 30‐day neurological outcomes in OHCA patients who survive more than 72 h. Maintaining higher hemoglobin levels in the days following ROSC could potentially improve short‐term mortality and neurological outcomes in OHCA patients.

## AUTHOR CONTRIBUTIONS

I‐Wei Ho contributed to the conception and design, interpretation of data, and drafted the manuscript. Ming‐Jen Kuo contributed to data acquisition and drafted the manuscript. Pai‐Feng Hsu, I‐Hsin Lee, Teh‐Fu Hsu, and Yenn‐Jiang Lin contributed to data acquisition. Chin‐Chou Huang contributed to the conception and design, data acquisition, data analysis, interpretation of data, and drafted and critically revised the manuscript. All authors gave final approval and agreed to be accountable for all aspects of the work, ensuring integrity and accuracy.

## CONFLICT OF INTEREST STATEMENT

The authors declare no conflict of interest.

## Supporting information

supplementary Figure 1.

## Data Availability

The data that support the findings of this study are available from the corresponding author upon reasonable request.
