# Supplementary figures and images for: The impacts of anemia burden on clinical outcomes in patients with out‐of‐hospital cardiac arrest
Source: Clin Cardiol. 2023 Oct 24;47(1):e24175. doi: 10.1002/clc.24175 (PMC10777437; doi:10.1002/clc.24175)

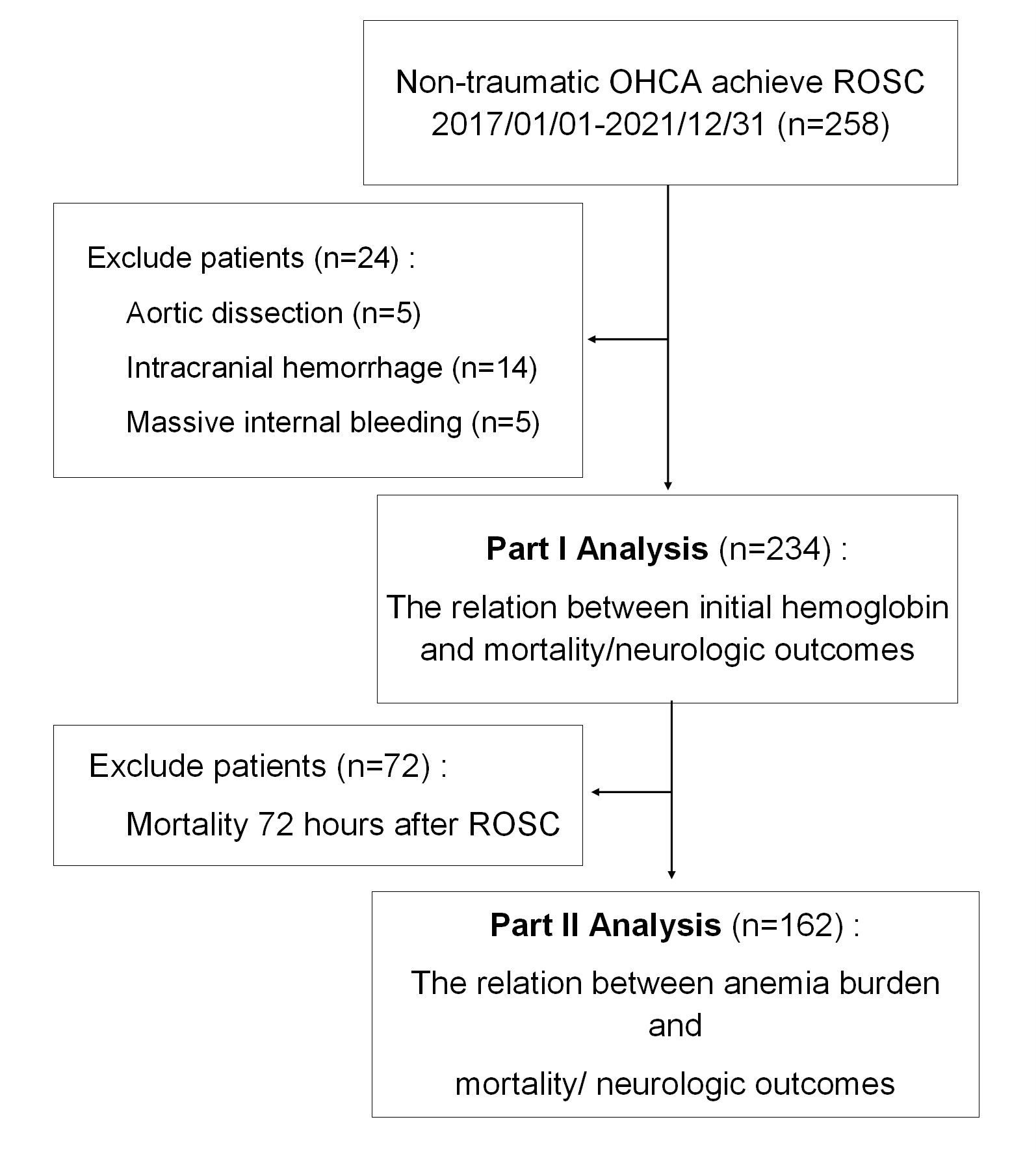

Supplement: Supplementary file 1 — supplementary Figure 1. [file CLC-47-e24175-s001.tif]
